# A diagnostic stewardship approach to prevent unnecessary testing of an enteric bacterial molecular panel

**DOI:** 10.1128/spectrum.02945-23

**Published:** 2023-10-30

**Authors:** Victoria L. Campodónico, Ann Hanlon, Michael W. Mikula, Jo-Anne Miller, Michael Gherna, Karen C. Carroll, Patricia J. Simner

**Affiliations:** 1 Division of Medical Microbiology, Department of Pathology, The Johns Hopkins University School of Medicine, Baltimore, Maryland, USA; 2 Division of Infectious Diseases, Department of Medicine, The Johns Hopkins University School of Medicine, Baltimore, Maryland, USA; Tainan Hospital, Ministry of Health and Welfare, Tainan,, Taiwan

**Keywords:** enteric bacterial panels, diagnostic stewardship, three-day rule, hard-stop, restriction override, hospital acquired diarrhea, quality improvement

## Abstract

After stool culture was replaced by an enteric bacterial panel (EBP) at the Johns Hopkins Hospital (JHH), many EBPs were ordered >3 days after admission (EBP >3 days) with low yield. To reduce unnecessary testing, a hard stop for ordering EBP >3 days was established with the option to override the restriction. The present study retrospectively evaluates the usefulness of overriding that restriction. A retrospective chart review was performed on all patients with an EBP >3 days at the JHH from November 2021 to October 2022. The override provided only one new diagnosis of *Salmonella* infection in a patient who developed diarrhea within 3 days of admission but was not tested by EBP until day 5. There were no positive EBPs >3 days in patients without diarrhea or who developed diarrhea >3 days after admission (diarrhea >3 days). Most patients with requests to override the restriction were also tested for *Clostridioides difficile*. All patients positive for *C. difficile* received treatment and their *C. difficile* positive test resulted >1 day before the EBP was run. There were no differences in yield in patients >65 years of age with significant comorbidities or associated with neutropenia, leukocytosis, human immunodeficiency virus status, or lactoferrin positivity. There were no positive tests in patients receiving stool softeners within the previous 48 hours of testing. Overriding the hard stop should not be approved in patients without diarrhea or with diarrhea >3 days, who received stool softeners within the previous 48 hours or have not been tested for more common causes of hospital-acquired diarrhea.

**IMPORTANCE:**

Testing for enteric bacterial pathogens in patients hospitalized for more than 3 days is almost always inappropriate. Our study validates the utility of the 3-day rule and the use of clinical decision support tools to decrease unnecessary testing of enteropathogenic bacteria other than *C. difficile*. Overriding the restriction was very low yield. Our study highlights the importance of diagnostic stewardship and further refines the criteria for allowing providers to override the restriction while monitoring the impact of the interventions.

## INTRODUCTION

Diarrhea that develops after 3 days of admission to the hospital is more likely associated with hospital-acquired infections (e.g., *Clostridioides difficile*, norovirus), enteral feeding, medications (e.g., antibiotics and other therapies) or underlying conditions than with enteric bacterial pathogens, which are usually community acquired (1, 2). Therefore, the 3-day rule, which states that clinicians should not order stool cultures after 3 days of patient admission, was established in the 1990s (3). With the transition to culture-independent methods, the same 3-day rule has since proven applicable to enteric bacterial molecular panels (4). Additionally, some clinical laboratories have implemented a hard-stop alert in electronic health record systems as a clinical decision support tool (CDST) to stop any stool culture or enteric bacterial molecular panel tests from being ordered for patients hospitalized for more than 3 days (5).

At the Johns Hopkins Hospital (JHH), molecular detection of enteric bacterial pathogens using the BD MAX enteric bacterial panel (EBP; BD Diagnostic Systems, Sparks, MD) replaced conventional stool culture, with the exception of *Aeromonas* culture, in 2016. For the following 3 years, up to 25% of the EBPs were ordered more than 3 days after patient admission with low yield (positivity rate 1.26% versus 8% for EBPs performed within 3 days of admission), with most positives (81%) occurring in patients whose diarrhea developed before or within 3 days of admission. Therefore, to reduce unnecessary testing, a computerized CDST (i.e., the hard-stop alert tool) was established in the electronic medical record (Epic, Verona, WI) in 2019. This CDST stopped any EBP ordered more than 3 days after patient hospitalization (EBP >3 days). The hard stop was implemented with a comment notifying the clinicians that they could contact the microbiology laboratory to get the restriction overridden if clinically indicated. Each request was reviewed by a Clinical Pathology Resident or a Microbiology Fellow to determine whether the EBP >3 days should be approved or denied at their discretion.

This quality improvement project was performed to retrospectively evaluate the usefulness of overriding the restriction to order EBP >3 days, to better determine criteria for approving or denying the override, and to develop tools to evaluate the impact of the interventions.

## MATERIALS AND METHODS

The BD MAX Extended EBP used in this study detects nucleic acids from *Salmonella* spp., *Campylobacter* spp. (*jejuni*/*coli*), *Shigella* spp./enteroinvasive *Escherichia coli* (EIEC), Shiga toxin-producing organisms (STEC, *Shigella dysenteriae*), enterotoxigenic *E. coli* (ETEC), *Yersinia enterocolitica, Plesiomonas shigelloides,* and *Vibrio* spp. (*vulnificus/parahaemolyticus/cholerae*).

A retrospective data search at JHH in Baltimore, Maryland, USA was performed to identify all patients with EBP >3 days ordered from November 2021 to October 2022. A retrospective chart review of all patients was performed to obtain information on the presence or absence of diarrhea, other diagnostic stool testing performed, laxative use, ordering department, presence or absence of neutropenia, leukocytosis, human immunodeficiency virus (HIV) infection, and elevated lactoferrin. The results from any positive test were also retrieved.

Based on JHH Medicine policies, this project, performed for quality improvement purposes, was not considered human subjects research and therefore did not require Institutional Review Board approval.

## RESULTS

Between November 2021 and October 2022, of 3,720 total EBPs ordered, 129 (3.5%) were requested after 3 days of patient admission (Table 1). Twenty-three (18%) of the requests for overriding the restriction were for patients without diarrhea, 65% of which were approved for testing, but no organisms were detected in any of them. Table S1 lists the reasons offered by providers as justification for ordering the EBP >3 days in patients without diarrhea. One hundred six (82%) EBP >3 days were ordered in patients with diarrhea. A total of 60 (57%) of these were for patients who developed diarrhea before or within 3 days of admission (diarrhea <3 days), but had their EBPs requested after the 3-day period, with 55 EBPs being approved and only 2 (4%) being positive, both for *Salmonella* spp. The positive results were obtained from a patient in their 70s with a history of *Salmonella* group D bacteremia on a previous recent admission and from a patient in their 90s with acute cholecystitis and negative blood cultures. The EBP >3 days of these two patients were ordered on days 4 and 5 after admission, respectively. Forty-six (43%) patients developed diarrhea after 3 days of admission (diarrhea >3 days), 35 (76%) of them had their EBP >3 days approved, and there were no positives (Table 1).

**TABLE 1:** Requests for overriding the restriction to order an EBP >3 days (*n* = 129)

	Start of diarrhea	Cancelled	Approved	Positive	Days of admission when test was ordered [mean (range)]
No diarrhea *n* = 23 (18%)		8 (35%)	15 (65%)	0	14 (4, 73)
Diarrhea *n* = 106 (82%)	< 3 days of admission *n* = 60 (57%)	5 (8%)	55 (92%)	2 (4%)^*a*^	9 (4, 44)
	> 3 days of admission *n* = 46 (43%)	11 (24%)	35 (76%)	0	46 (4, 180)

^*a*^ Tests ordered on days 4 and 5 of admission, respectively. Both positive for *Salmonella* spp.

Of the 129 orders requested after 3 days of admission, 121 (94%) had at least one other test ordered concurrently with the EBP >3 days (Table 2). Seventy-four (57%) patients were tested for enteric parasites [36 (28%) for microsporidia, 66 (51%) for protozoan parasites [*Giardia duodenalis, Cryptosporidium* spp. (*C. parvum* and *C. hominis*) and *Entamoeba histolytica*], 44 (34%) for ova and parasites (O&P), 5 (4%) for *Cyclospora/Cystoisospora*] with no organisms detected in any of those tests. Twenty-six (20%) and 37 (29%) patients were tested for rotavirus and norovirus, respectively, with one patient being positive for each of them. Both of these patients had developed diarrhea <3 days. Additionally, 32 (25%) patients were tested for adenovirus, 11 (9%) for *Helicobacter pylori*, 2 (2%) for *Mycobacterium* spp., and 4 (3%) for *Aeromonas* spp. with no organisms detected (Table 2). *C. difficile* testing was ordered in 106 (82%) patients, 16 (15%) patients without diarrhea, and 15 (14%) patients receiving stool softeners. All 17 (16%) *C. difficile* positive tests resulted at least 1 day before the EBP >3 days was run and they all prompted antibiotic treatment (Table 3). Overall, there were 19 (15%) EBP >3 days ordered in patients receiving stool softeners, 13 (68%) were approved, and there were no positives (Table 3).

**TABLE 2:** Other stool tests ordered concurrently with the EBP >3 days

Other stool tests ordered [*n* (*n* positive)]												
	Start of diarrhea	*C. difficile*	Microsporidia	Protozoan NAT	O&P	*Cyclospora / Cystoisospora*	Rotavirus	Adenovirus	Norovirus	*Helicobacter pylori*	Mycobacteria	*Aeromonas* spp.
No diarrhea *n* = 23 (18%)		16 (4)	5 (0)	9 (0)	5 (0)	1 (0)	1 (0)	1 (0)	1 (0)	3 (0)	0	0
Diarrhea *n* = 106 (82%)	<3 days of admission *n* = 60 (57%)	51 (8)	16 (0)	33 (0)	20 (0)	2 (0)	16 (1)	15 (0)	21 (1)	5 (0)	2 (0)	1 (0)
	>3 days of admission *n* = 46 (43%)	39 (5)	15 (0)	24 (0)	19 (0)	2 (0)	9 (0)	16 (0)	15 (0)	3 (0)	0	3 (0)
Total		106 (17)	36 (0)	66 (0)	44 (0)	5 (0)	26 (1)	32 (0)	37 (1)	11 (0)	2 (0)	4 (0)

**TABLE 3:** Patients who underwent *C. difficile* testing and/or were receiving stool softeners

	Start of diarrhea	Stool softener	*C. difficile*			EBP approved [total *n* (*n* positive)]
			Negative (*n* = 89)	Positive (*n* = 17)	Not tested (*n* = 23)	
No diarrhea *n* = 23 (18%)		No (*n* = 13)	6	3	3	7 (0)
		Yes (*n* = 10)	6	1	4	8 (0)
Diarrhea *n* = 106 (82%)	<3 days of admission *n* = 60 (57%)	No (*n* = 55)	39	7	9	52 (2)
		Yes (*n* = 5)	4	1	0	3 (0)
	>3 days of admission *n* = 46 (43%)	No (*n* = 42)	32	4	6	33 (0)
		Yes (*n* = 4)	2	1	1	2 (0)

There were 13 (10%) patients with neutropenia at the time the EBP >3 days was ordered, and one of them who developed diarrhea >3 days was positive for *C. difficile* (Table 4). Leukocytosis was present in 36 (28%) patients, 3 (2%) were positive for *C. difficile* (1 in a patient without diarrhea and 2 in patients who developed diarrhea <3 days), and 1 (1%) was positive for *Salmonella* spp. Additionally, nine (7%) patients living with HIV had an EBP >3 days ordered and two were positive for *C. difficile* (one in a patient without diarrhea and one in a patient with diarrhea <3 days). Only 39 patients were tested for the presence of elevated lactoferrin and 28 (72%) were positive. Three (11%) of the lactoferrin-positive patients did not have diarrhea with one being positive for *C. difficile*. An additional 15 (54%) patients who were lactoferrin positive had developed diarrhea <3 days and three had an identified pathogen (*C. difficile, Salmonella* spp., and rotavirus). Finally, of the 10 patients with diarrhea >3 days and lactoferrin positive, 2 were positive for *C. difficile*.

**TABLE 4:** Additional criteria that could be considered for approving an EBP >3 days

	Start of diarrhea		Neutropenia		Leukocytosis		Living with HIV		Positive Lactoferrin	
			No (*n* = 116)	Yes (*n* = 13)	No (*n* = 93)	Yes (*n* = 36)	No (*n* = 120)	Yes (*n* = 9)	No (*n* = 11)	Yes (*n* = 28)
No diarrhea *n* = 23 (18%)		No organism (*n* = 19)	19 (16%)	0	12 (13%)	7 (19%)	17 (14%)	2 (22%)	0	2 (7%)
		*C. difficile* (*n* = 4)	4 (3%)	0	3 (3%)	1 (3%)	3 (2%)	1 (11%)	0	1 (3.5%)
Diarrhea *n* = 106 (82%)	<3 days of admission *n* = 60 (57%)	No organism (*n* = 48)	44 (39%)	4 (31%)	35 (38%)	13 (36%)	45 (38%)	3 (33%)	6 (55%)	12 (43%)
		*C. difficile* (*n* = 8)	8 (7%)	0	6 (7%)	2 (6%)	7 (6%)	1 (11%)	1 (9%)	1 (3.5%)
		*Salmonella* (*n* = 2)	2 (2%)	0	1 (1%)	1 (3%)	2 (2%)	0	0	1 (3.5%)
		Norovirus (*n* = 1)	1 (1%)	0	1 (1%)	0	0	1 (11%)	0	0
		Rotavirus (*n* = 1)	1 (1%)	0	1 (1%)	0	1 (1%)	0	0	1 (3.5%)
	>3 days of admission *n* = 46 (43%)	No organism (*n* = 41)	33 (28%)	8 (61%)	29 (31%)	12 (33%)	40 (33%)	1 (11%)	4 (36%)	8 (29%)
		*C. difficile* (*n* = 5)	4 (3%)	1 (8%)	5 (5%)	0	5 (4%)	0	0	2 (7%)

There were 20 (16%) EBP >3 days ordered in patients >65 years old with significant comorbidities causing permanently altered organ function (i.e., cirrhosis, end-stage renal disease, chronic obstructive pulmonary disease, active inflammatory bowel disease, and leukemia). Sixteen of these EBP >3 days were approved and there were no positives.

Most EBP >3 days overrides were requested by the internal medicine department (29%), followed by gastroenterology (22%), and infectious diseases (19%). All departments ordered at least one test in patients without diarrhea and infectious diseases was the department with the lowest percentage of canceled tests (Table 5). Additionally, most cancellations were due to the absence of diarrhea (33%) or the use of stool softeners (21%). None of the patients with EBP >3 days not approved had a follow-up diagnosis of bacterial enteric pathogens within the same diarrheic episode.

**TABLE 5:** Clinical departments requesting to override the restriction to order an EBP >3 days

Ordering department (*n* = 129)	*n* (%)	No diarrhea		Diarrhea started <3 days of admission		Diarrhea started >3 days of admission		Total cancelled	Total approved	Positive
		Cancelled	Approved	Cancelled	Approved	Cancelled	Approved			
Internal medicine	38 (29)	2 (33%)	4 (67%)	1 (6%)	17 (94%)	5 (36%)	9 (64%)	8 (21%)	30 (79%)	1
GI^*a*^	29 (22)	1 (20%)	4 (80%)	1 (8%)	12 (92%)	4 (36%)	7 (64%)	6 (21%)	23 (79%)	0
Infectious diseases	25 (19)	0	3 (100%)	1 (6%)	17 (94%)	1 (25%)	3 (75%)	2 (8%)	23 (92%)	0
General pdiatrics	11 (9)	1 (100%)	0	1 (20%)	4 (80%)	0	5 (100%)	2 (18%)	9 (81%)	0
Intensive care	9 (7)	1 (25%)	3 (75%)	0	2 (100%)	1 (33%)	2 (67%)	2 (22%)	7 (78%)	1
Oncology	9 (7)	2 (67%)	1 (33%)	0	1 (100%)	0	5 (100%)	2 (22%)	7 (78%)	0
Surgery	8 (6)	1 (100%)	0	1 (33%)	2 (67%)	0	4 (100%)	2 (25%)	6 (75%)	0

^*a*^ GI: gastroenterology.

## DISCUSSION

Consistent with previous studies, this retrospective study performed in an academic tertiary-care hospital demonstrated that the use of the hard-stop alert tool to guide clinicians on the appropriate stool test for patients with diarrhea who had been hospitalized for more than 3 days significantly reduced unnecessary EBP >3 days testing, from 25% to 3.5% (5). The hard-stop was implemented giving the clinicians the option to call the laboratory to override the restriction to prevent patient harm and clinician dissatisfaction. Overriding the restriction involved evaluation by the Clinical Pathology Resident or Microbiology Fellow, which required time to review the patient’s chart and contact the providers to discuss the requests. Furthermore, there were no clear approval guidelines in place and the knowledge to determine whether a test was appropriately ordered could vary significantly between different reviewers. Therefore, this quality improvement project was developed to evaluate the usefulness of overriding the restriction to order the EBP >3 days and to establish criteria for approving or denying the test.

Overall, overriding the hard-stop provided only one new diagnosis (0.95%) in a patient whose EBP was ordered after 3 days of admission but who had developed diarrhea within 3 days of hospitalization. This patient had a diagnosis of acute cholecystitis, which can occur in 2% of *Salmonella* Typhi infections and rarely in nontyphoidal *Salmonella* infections (6). Additionally, approving the EBP >3 days in patients without diarrhea did not provide any positive results, confirming prior recommendations that testing patients without diarrhea for enteric bacterial pathogens should not be performed (7). EBPs detect DNA and not necessarily viable organisms, and enteropathogens can be excreted by persons without diarrhea for weeks after they have recovered from diarrheal episodes experienced many weeks earlier (8). Furthermore, asymptomatic carriage of known enteric pathogens, such as *Campylobacter* spp., *Salmonella* spp., *G. duodenalis,* and *C. parvum* has been described, particularly in low-resource settings (8 – 11). Therefore, a positive result may not indicate an active infection but the identification of carriers. Additionally, the optimal specimen for laboratory diagnosis of infectious diarrhea is a diarrheal stool sample (i.e., a sample that takes the shape of the container), although molecular techniques are less dependent than culture on the quality of the specimen (7). Another factor to take into consideration when testing for enteric bacterial pathogens in patients without diarrhea using EBPs is reimbursement as the Medicare Indications and Limitations of Coverage criteria may require the presence of diarrhea to consider an EBP medically reasonable and necessary (https://www.cms.gov/medicare-coverage-database/view/lcd.aspx?lcdid=38229&ver=22). However, based on the 2017 Infectious Diseases Society of America (IDSA) Practice Guidelines for the Diagnosis and Management of Infectious Diarrhea exceptions to this rule would be when there is a clinical suspicion of enteric fever where diarrhea is uncommon, but shedding of *Salmonella* spp. in the stool could be detected by a multiplex molecular diagnostic panel; and to test for *Y. enterocolitica* in people with persistent abdominal pain (especially school-aged children with right lower quadrant pain mimicking appendicitis who may have mesenteric adenitis), and in people with fever at epidemiologic risk for yersiniosis (7).

Overriding the restriction in patients who developed diarrhea >3 days did not lead to the identification of any pathogens detected by the EBP. Some authors have advocated for a modified 3-day rule, where the presence of certain criteria, such as neutropenia, leukocytosis, HIV positivity, and patients older than 65 years of age with significant comorbidities causing permanently altered organ function, would allow test approval (12 – 14). However, none of the 16 patients who underwent EBP >3 days testing meeting at least one of those criteria within our cohort had a positive EBP >3 days, suggesting that each of those conditions alone should not trigger approval of testing, although due to the small sample size, this may warrant further investigation. Additionally, although lactoferrin is a marker of intestinal inflammation, it is nonspecific, and, in our study, the presence of positive lactoferrin did not seem to correlate with the presence of acute infectious diarrhea (*P* > 0.05, Fisher exact test) and therefore, as recommended by IDSA, it should not be used to establish the cause of acute infectious diarrhea (7).

More than half of the patients with EBP >3 days ordered had at least one diagnostic test for intestinal parasites performed with very low yield, which is consistent with previous studies (5, 15) and the prevalence of enteric parasites in our patient population, which suggests that many of these parasitic studies were most probably inappropriately ordered. Current guidelines recommend testing for parasites only in the context of a possible outbreak of diarrheal illness, in immunocompromised patients with diarrhea, especially those with moderate and severe primary or secondary immune deficiencies, or when there are risk factors for parasitic infections, such as exposure of a traveler to untreated water, swimming in freshwater, traveling to endemic areas, and when intestinal symptoms persist for more than seven days (7, 16 – 18). Additionally, several studies have suggested that a “3-day rule” should also be considered for stool parasitological studies, particularly for O&P exams (15, 19, 20). We did not evaluate the positivity rate of all the parasitological studies performed after 3 days of admission at JHH. This might be another area to evaluate diagnostic stewardship efforts and future quality improvement projects.

*C. difficile* is the primary pathogen associated with antibiotic-associated colitis, and it is the most frequent cause of infectious nosocomial diarrhea (21 – 23). Therefore, most patients (82%) admitted to the hospital for more than 3 days with a request to override the restriction to test for EBP >3 days were concurrently tested for *C. difficile*. Interestingly, even though there were policies in place to ensure testing only on patients at risk of *C. difficile* infection (CDI), in terms of a best practice alert (i.e., a hard-stop) to prevent testing of patients who had received a laxative within the previous 48 hours or who did not have diarrhea (24), 15 (14%) *C. difficile* tests were performed in patients receiving stool softeners and 16 (15%) patients with no diarrhea were tested. Additionally, all patients who tested positive for *C. difficile* received treatment for CDI suggesting that they were all considered clinically significant. While IDSA *C. difficile* guidelines recommend only testing patients likely to have *C. difficile* disease, which includes not routinely performing testing on stool from a patient who does not have clinically significant diarrhea or who has received a laxative within the previous 48 hours, such a recommendation is labeled as weak and based on very low-quality evidence (25). Furthermore, a recent study showed that there were no differences in the rates or severity of CDI among patients based on whether or not they had received laxatives within the previous 48 hours (26). Although hospitals are encouraged to follow the IDSA guidelines for *C. difficile* testing and have diagnostic stewardship efforts in place, there should be allowances for exceptions to the rules based on clinical judgment (27). Unfortunately, guidelines for multiplex molecular testing for enteric bacterial pathogens other than *C. difficile* and the use of laxatives have not been well established. A recent study evaluating the appropriateness and diagnostic stewardship opportunities of multiplex gastrointestinal molecular testing considered EBP testing inappropriate when there was laxative use in the preceding 48 hours of sample collection. However, there were no statistically significant differences in positive test results between patients who had received laxatives and those who had not, and no discussion about the clinical significance of the positive results was provided (28). Nevertheless, based on the Medicare coverage database, coverage for multiplex EBPs might be denied when the tests are performed in patients who had received laxatives in the prior 48 hours, excluding those with signs of severe disease (https://www.cms.gov/medicare-coverage-database/view/lcd.aspx?lcdid=39226&ver=7). Therefore, it is worth considering the use of laxatives within 48 hours prior to testing as a factor for restricting EBP >3 days testing.

**Fig 1:**
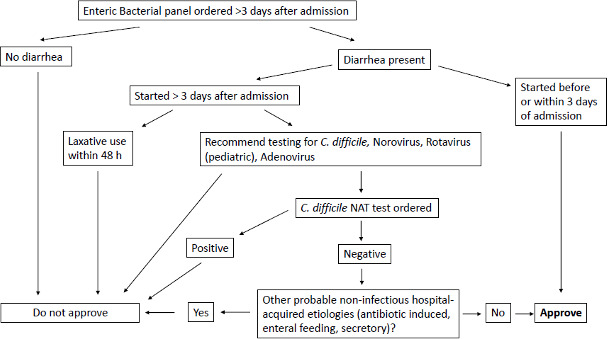
Algorithm for an EBP >3 days override approval. Following the new algorithm for EBP >3 days override approval, EBP >3 days will be denied in patients with no diarrhea. If a patient develops diarrhea before or within 3 days of admission, the request will be approved. If diarrhea develops after 3 days of admission and the patient is receiving laxatives, it will be recommended to stop them for 48 hours, and then reconsider if the test is still needed. Testing for more common causes of nosocomial infections will also be recommended. Then, if no other non-infectious causes of nosocomial diarrhea are probable, the EBP >3 days will be approved.

With all the information we obtained from our retrospective analysis, we developed an algorithm for approval of the requests for overriding the restriction for EBP >3 days (Fig. 1). Absence of diarrhea will be the first criterion for denying the test, which, based on our current data, could result in an 18% reduction of approved tests. If the patient developed diarrhea before or within 3 days of admission, but the request for the EBP was delayed for whatever reason, we will approve it, consistent with our two positive *Salmonella* results, which were both from patients who developed diarrhea <3 days. In cases where diarrhea developed after 3 days of admission, if the patient is receiving laxatives, we will recommend to stop them for 48 hours, then reconsider if the test is still needed. We will also recommend testing for more common causes of nosocomial infections, such as *C. difficile*, norovirus, adenovirus, and rotavirus (for pediatric patients), with particular focus on CDI. Once these tests resulted and if negative, we will discuss the possibility of other non-infectious causes of nosocomial diarrhea and if those are of low probability, we will then approve the EBP >3 days. Additionally, we will implement the use of a REDCap (Research Electronic Data Capture) questionnaire that will be completed by the Clinical Pathology Resident or Microbiology Fellow reviewing each case. The questionnaire will collect similar information to the one obtained for this study, and it will allow us to monitor the impact of the interventions in real-time. Furthermore, we will continue monitoring which departments are requesting the overrides and perform targeted education if needed (i.e., for departments ordering requests from patients without diarrhea and high percentages of canceled requests).

There are several limitations to this study. This was a single-site study at an academic tertiary-care medical center in a developed country; therefore, these results may not be generalizable to small community hospitals or to developing countries where enteric bacterial pathogens are more prevalent. Additionally, our algorithm for overriding the restriction to order EBP >3 days requires the review of patients’ records, and other institutions may not have personnel available to perform this task. Also, we were not able to determine the actual reasons why an override request was approved or rejected because there were no records available with that information. Furthermore, we did not evaluate specific clinical outcomes, like antibiotic use, or the cost savings associated with reducing inappropriate EBP >3 days testing. However, following the proposed algorithm, EBP >3 days testing would have been reduced by at least 42% if none of the patients without diarrhea, *C. difficile*, norovirus, and rotavirus detection or receiving stool softeners were tested in this study. Decreased testing could have resulted in significantly reduced costs.

Our quality improvement project validates the utility of the 3-day rule and the clinical decision support tools to decrease unnecessary testing for detection of enteropathogenic bacteria other than *C. difficile* in patients admitted to the hospital for more than 3 days. While overriding the restriction to order the EBP >3 days was very low yield and required substantial time, it is important to allow providers to order the test if they consider it medically reasonable and necessary. Hopefully, the new algorithm will provide consistent education regarding best practices. Additionally, it will ensure that every case is approved or denied using the same criteria regardless of who is reviewing the request.

## SUPPLEMENTAL MATERIAL

Table S1 spectrum.02945-23-s0001.docx Criteria to order EBP >3 days.

ASM does not own the copyrights to Supplemental Material that may be linked to, or accessed through, an article. The authors have granted ASM a non-exclusive, world-wide license to publish the Supplemental Material files. Please contact the corresponding author directly for reuse.

## References

[1] Burnham C-AD , Carroll KC . 2013. Diagnosis of Clostridium difficile infection: an ongoing conundrum for clinicians and for clinical laboratories. Clin Microbiol Rev 26:604–630. doi:10.1128/CMR.00016-13 23824374 PMC3719497

[2] Polage CR , Solnick JV , Cohen SH . 2012. Nosocomial diarrhea: evaluation and treatment of causes other than Clostridium difficile. Clin Infect Dis 55:982–989. doi:10.1093/cid/cis551 22700831 PMC3657522

[3] Valenstein P , Pfaller M , Yungbluth M . 1996. The use and abuse of routine stool Microb abuse of routine stool microbiology: a college of american patn Pathologists Q-probes study of 601 institutions. Arch Pathol Lab Med 120:206–211. 8712901

[4] Hitchcock MM , Gomez CA , Banaei N . 2018. Low yield of FilmArray GI panel in hospitalized patients with diarrhea: an opportunity for diagnostic stewardship intervention. J Clin Microbiol 56:e01558-17. doi:10.1128/JCM.01558-17 29237784 PMC5824048

[5] Nikolic D , Richter SS , Asamoto K , Wyllie R , Tuttle R , Procop GW . 2017. Implementation of a clinical decision support tool for stool cultures and parasitological studies in hospitalized patients. J Clin Microbiol 55:3350–3354. doi:10.1128/JCM.01052-17 28954902 PMC5703801

[6] McCarron B , Love WC . 1997. Acalculous nontyphoidal salmonellal cholecystitis requiring surgical intervention despite ciprofloxacin therapy: report of three cases. Clin Infect Dis 24:707–709. doi:10.1093/clind/24.4.707 9145746

[7] Shane AL , Mody RK , Crump JA , Tarr PI , Steiner TS , Kotloff K , Langley JM , Wanke C , Warren CA , Cheng AC , Cantey J , Pickering LK . 2017. Infectious diseases society of America clinical practice guidelines for the diagnosis and management of infectious diarrhea. Clin Infect Dis 65:1963–1973. doi:10.1093/cid/cix959 29194529 PMC5848254

[8] Levine MM , Robins-Browne RM . 2012. Factors that explain excretion of Enteric pathogens by persons without diarrhea. Clin Infect Dis 55 Suppl 4:S303–11. doi:10.1093/cid/cis789 23169942 PMC3502317

[9] Amour C , Gratz J , Mduma E , Svensen E , Rogawski ET , McGrath M , Seidman JC , McCormick BJJ , Shrestha S , Samie A , et al., Etiology, Risk Factors, and Interactions of Enteric Infections and Malnutrition and the Consequences for Child Health and Development Project (MAL-ED) Network Investigators . 2016. Epidemiology and impact of campylobacter infection in children in 8 low-resource settings: results from the MAL-ED study. Clin Infect Dis 63:1171–1179. doi:10.1093/cid/ciw542 27501842 PMC5064165

[10] Rogawski ET , Bartelt LA , Platts-Mills JA , Seidman JC , Samie A , Havt A , Babji S , Trigoso DR , Qureshi S , Shakoor S , et al. . 2017. Determinants and impact of Giardia infection in the first 2 years of life in the MAL-ED birth cohort. J Pediatric Infect Dis Soc 6:153–160. doi:10.1093/jpids/piw082 28204556 PMC5907871

[11] Rogawski ET , Guerrant RL . 2017. “The burden of enteropathy and "subclinical" infections”. Pediatr Clin North Am 64:815–836. doi:10.1016/j.pcl.2017.03.003 28734512 PMC5523808

[12] Bauer TM . 2001. Derivation and validation of guidelines for stool cultures for enteropathogenic bacteria other than Clostridium difficile in hospitalized adults. JAMA 285:313. doi:10.1001/jama.285.3.313 11176841

[13] Beal SG , Velez L , Tremblay EE , Toffel S , Rand KH . 2018. “The "3-day rule" for stool tests may not apply when using PCR panels”. J Clin Microbiol 56:e02012-17. doi:10.1128/JCM.02012-17 29581319 PMC5869825

[14] Seyler L , Lalvani A , Collins L , Goddard L , Bowler ICJW . 2007. Safety and cost savings of an improved three-day rule for stool culture in hospitalised children and adults. J Hosp Infect 67:121–126. doi:10.1016/j.jhin.2007.07.010 17900758

[15] Morris AJ , Wilson ML , Reller LB . 1992. Application of rejection criteria for stool ovum and parasite examinations. J Clin Microbiol 30:3213–3216. doi:10.1128/jcm.30.12.3213-3216.1992 1452704 PMC270631

[16] Golfeyz S , Haviland A , Burger A . 2021. Things we do for no reason (TM): ova and parasite testing in patients with acute diarrhea arising during hospitalization. J Hosp Med 16:236–238. doi:10.12788/jhm.3498 33734981

[17] Riddle MS , DuPont HL , Connor BA . 2016. ACG clinical guideline: diagnosis, treatment, and prevention of acute diarrheal infections in adults. Am J Gastroenterol 111:602–622. doi:10.1038/ajg.2016.126 27068718

[18] Thielman NM , Guerrant RL . 2004. Acute infectious diarrhea. N Engl J Med 350:38–47. doi:10.1056/NEJMcp031534 14702426

[19] Guerrant RL , Van Gilder T , Steiner TS , Thielman NM , Slutsker L , Tauxe RV , Hennessy T , Griffin PM , DuPont H , Sack RB , Tarr P , Neill M , Nachamkin I , Reller LB , Osterholm MT , Bennish ML , Pickering LK , Infectious Diseases Society of America . 2001. Practice guidelines for the management of infectious diarrhea. Clin Infect Dis 32:331–351. doi:10.1086/318514 11170940

[20] Siegel DL , Edelstein PH , Nachamkin I . 1990. Inappropriate testing for diarrheal diseases in the hospital. JAMA 263:979. doi:10.1001/jama.1990.03440070067034 2299766

[21] Asha NJ , Tompkins D , Wilcox MH . 2006. Comparative analysis of prevalence, risk factors, and molecular epidemiology of antibiotic-associated diarrhea due to Clostridium difficile, Clostridium Perfringens, and Staphylococcus aureus. J Clin Microbiol 44:2785–2791. doi:10.1128/JCM.00165-06 16891493 PMC1594656

[22] Humphries RM , Linscott AJ . 2015. Practical guidance for clinical microbiology laboratories: diagnosis of bacterial gastroenteritis. Clin Microbiol Rev 28:3–31. doi:10.1128/CMR.00073-14 25567220 PMC4284301

[23] McFarland LV . 1995. Epidemiology of infectious and iatrogenic nosocomial diarrhea in a cohort of general medicine patients. Am J Infect Control 23:295–305. doi:10.1016/0196-6553(95)90060-8 8585641

[24] Mizusawa M , Small BA , Hsu Y-J , Sharara SL , Advic E , Kauffman C , Milstone AM , Feldman L , Pahwa AK , Trivedi JB , Landrum MB , Maragakis LL , Carroll KC , Cosgrove SE , Rock C . 2019. Prescriber behavior in Clostridioides difficile testing: A 3-hospital diagnostic stewardship intervention. Clin Infect Dis 69:2019–2021. doi:10.1093/cid/ciz295 31125399

[25] McDonald LC , Gerding DN , Johnson S , Bakken JS , Carroll KC , Coffin SE , Dubberke ER , Garey KW , Gould CV , Kelly C , Loo V , Shaklee Sammons J , Sandora TJ , Wilcox MH . 2018. Clinical practice guidelines for Clostridium difficile infection in adults and children: 2017 update by the infectious diseases society of America (IDSA) and society for healthcare epidemiology of America (SHEA). Clin Infect Dis 66:e1–e48. doi:10.1093/cid/cix1085 29462280 PMC6018983

[26] White NC , Mendo-Lopez R , Papamichael K , Cuddemi CA , Barrett C , Daugherty K , Pollock N , Kelly CP , Alonso CD . 2020. Laxative use does not preclude diagnosis or reduce disease severity in clostridiodes difficile infection. Clin Infect Dis 71:1472–1478. doi:10.1093/cid/ciz978 31584632 PMC7486840

[27] Rock C , Maragakis LL . 2020. Diagnostic stewardship for clostridiodes difficile testing: from laxatives to diarrhea and beyond. Clin Infect Dis 71:1479–1480. doi:10.1093/cid/ciz982 31584627

[28] O’Neal M , Murray H , Dash S , Al-Hasan MN , Justo JA , Bookstaver PB . 2020. Evaluating appropriateness and diagnostic stewardship opportunities of multiplex polymerase chain reaction gastrointestinal testing within a hospital system. Ther Adv Infect Dis 7:2049936120959561. doi:10.1177/2049936120959561 33014363 PMC7513010

