# Supplementary material for: A diagnostic stewardship approach to prevent unnecessary testing of an enteric bacterial molecular panel
Source: Microbiol Spectr. 2023 Oct 30;11(6):e02945-23. doi: 10.1128/spectrum.02945-23 (PMC10715171; doi:10.1128/spectrum.02945-23)
Supplement: Table S1 — Criteria to order EBP >3 days. [file spectrum.02945-23-s0001.docx]

**Table S1. Clinical criteria used to order EBP >3d in patients without diarrhea**

| **Clinical criteria used to order EBP >3d in patients without diarrhea** |
| --- |
| Hematochezia, melena or blood in stool  Nausea, vomiting and abdominal pain (with constipation)  Guillain Barré Syndrome (*Campylobacter*?)  Constipation (sample obtained by enema) ​  Diverticulitis ​  Findings suggestive of colitis on CT scan and fever ​  Rhabdomyolysis (Shiga toxin producing organisms?) ​  CT showed colitis versus pancreatitis ​  Borborygmi ​  Fever  Unknown |
